# Radiobiological Optimization in Lung Stereotactic Body Radiation Therapy: Are We Ready to Apply Radiobiological Models?

**DOI:** 10.3389/fonc.2017.00321

**Published:** 2018-01-08

**Authors:** Marco D’Andrea, Silvia Strolin, Sara Ungania, Alessandra Cacciatore, Vicente Bruzzaniti, Raffaella Marconi, Marcello Benassi, Lidia Strigari

**Affiliations:** ^1^Laboratory of Medical Physics and Expert Systems, Regina Elena National Cancer Institute, Rome, Italy

**Keywords:** lung neoplasms, stereotactic body radiotherapy, radiobiological modeling, tumor control probability, normal tissue complication probability

## Abstract

Lung tumors are often associated with a poor prognosis although different schedules and treatment modalities have been extensively tested in the clinical practice. The complexity of this disease and the use of combined therapeutic approaches have been investigated and the use of high dose-rates is emerging as effective strategy. Technological improvements of clinical linear accelerators allow combining high dose-rate and a more conformal dose delivery with accurate imaging modalities pre- and during therapy. This paper aims at reporting the state of the art and future direction in the use of radiobiological models and radiobiological-based optimizations in the clinical practice for the treatment of lung cancer. To address this issue, a search was carried out on PubMed database to identify potential papers reporting tumor control probability and normal tissue complication probability for lung tumors. Full articles were retrieved when the abstract was considered relevant, and only papers published in English language were considered. The bibliographies of retrieved papers were also searched and relevant articles included. At the state of the art, dose–response relationships have been reported in literature for local tumor control and survival in stage III non-small cell lung cancer. Due to the lack of published radiobiological models for SBRT, several authors used dose constraints and models derived for conventional fractionation schemes. Recently, several radiobiological models and parameters for SBRT have been published and could be used in prospective trials although external validations are recommended to improve the robustness of model predictive capability. Moreover, radiobiological-based functions have been used within treatment planning systems for plan optimization but the advantages of using this strategy in the clinical practice are still under discussion. Future research should be directed toward combined regimens, in order to potentially improve both local tumor control and survival. Indeed, accurate knowledge of the relevant parameters describing tumor biology and normal tissue response is mandatory to correctly address this issue. In this context, the role of medical physicists and the AAPM in the development of radiobiological models is crucial for the progress of developing specific tool for radiobiological-based optimization treatment planning.

## Introduction

The efficacy of radiation therapy for lung tumors lies in delivering large radiation doses in schedules and treatment modalities that all have the same goal of tumor control while sparing normal tissue from excessive toxicity. Indeed, the technological improvements of clinical linear accelerators nowadays allow combining high dose-rate and a more conformal dose delivery with an accurate image modality pre- and during therapy. This has encouraged the use of severe treatment schedules with doses per fraction larger than 10 Gy and up to 20–30 Gy for non-small cell lung cancer (NSCLC), the most frequent type of lung cancer. The linear quadratic (LQ) model has been widely used for predicting tumor control and toxicity after conventional radiotherapy (1). Unfortunately, the prolongation of the overall treatment time beyond 4–5 weeks renders radiotherapy less effective due to the increased proliferation of tumor cells in particular for NSCLC. In order to improve patient outcome, stereotactic body radiotherapy (SBRT) or stereotactic ablative body radiotherapy (SABR) has been adopted overtime.

In particular, large fractions in short overall times allow an increase of biologically effective dose (BED) expressed in Gy_10_ while maintaining the BED for normal lung tissue (expressed in Gy_3_) under the commonly accepted constraints (1). At these dose levels, the radiobiological appropriateness and robustness of the models and the dose constrains adopted for conventional fractionation are under discussion, stimulating researchers to conduct pre-clinical and clinical studies. Case series and prospective phase I–II studies have consistently reported high rates of local control (87–95%) and overall survival (65–76%) at 2 to 3 years when SBRT is compared to conventional RT, while randomized clinical trials (RCTs) are still ongoing (2, 3).

To optimize cancer treatment protocols, normal tissue complication probability (NTCP) and tumor control probability (TCP) models have been used (4). Accurate knowledge of the relevant parameters describing tumor biology and normal tissue response is mandatory to correctly address this issue. Radiobiological knowledge can be implemented either adopting a forward “try and check” approach or using an inverse planning optimization strategy via suitably designed cost functions (5).

However, at the state of the art, very few commercial treatment planning systems (TPSs) include biologically based optimization, and each TPS is based on different models for plan optimization (6–8). In particular, the optimization strategies classified as “radiobiological” include metrics such as equivalent uniform dose (EUD) for tumors (9, 10), generalized Equivalent Uniform Biological Effective Dose (11), gEUBED for normal tissues (12), mean lung dose (MLD) (13), NTCP and TCP models and the “uncomplicated tumor local control probability” (14, 15). Generally, in the case of radiobiological-based planning, the objective functions contain radiobiological indexes, often used in addition to dose/volume constraints.

The purpose of this paper is to describe the state of the art and future directions in the use of radiobiological models to describe/predict dose–effect relationship as well as in the SBRT treatment plan optimization of lung cancer in clinical practice.

## Materials and Methods

### Studies Selection

The main aim of our search was to recover and reanalyze papers focusing on radiobiological models for describing/predicting dose–effect relationship as well as for the biologically based SBRT plan optimization. Thus, we performed a literature search on PubMed using the search approach reported in Supplementary Material. The date of the last search was 1 October 2017. Full articles were retrieved when the abstract was considered relevant and only papers published in English were contemplated. The bibliographies of retrieved papers and reviews were also searched to identify other relevant articles to be included.

Moreover, we completed the search using the following terms: “SBRT,” “SABR,” “radiation effects,” “toxicity,” “tolerance,” “rib,” “chest wall,” “vessel,” “bronchi,” ”brachial,” ”esophageal,” ”lung,” “gEUD,” and “biological optimization,” from 2000 up to September 2017.

The PRISMA methodology was used for study selection based on the following criteria. Two authors independently reviewed titles and abstracts for the inclusion and in case of controversial judgment, a third author evaluated the papers.

**Figure 1:**
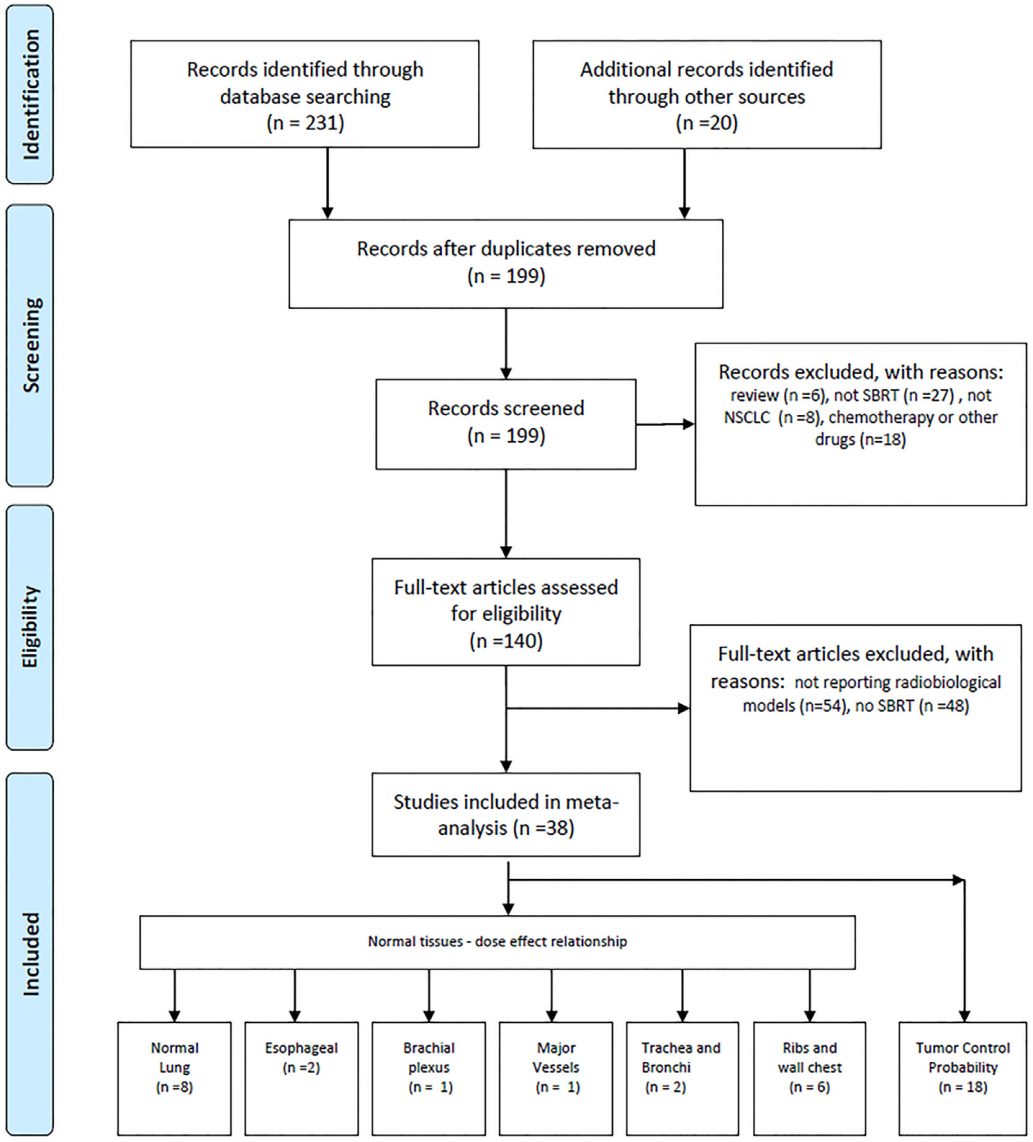
PRISMA-based methodology was used for study selection.

Papers were considered eligible when they reported data, tables, graphs/figures on dose–effect relationship or models developed for SBRT treatment. Papers reporting dose–effect relationship or models for chemotherapeutic treatments or receiving injection/administration of other drug in combination or subsequent to SBRT have been excluded. More details are shown in Figure 1.

### Radiobiological Models and BED Calculation

When several models are reported for the same endpoint a graphical comparison is reported.

Of note, the LQ model is the most used model adopted for conventional fractionation with only the basic assumptions that lung tumor α/β ratio is 10 Gy while α/β ratio for radiation pneumonitis (RP) and other late complications is 3 Gy, that the intrinsic radio-sensitivity of tumor cells is 0.35 ln/Gy, that no tumor repopulation occurs within 2 weeks, and that the model is sound up to 23 Gy per fraction (1).

When multiple models are available for a given endpoint, dose–effect relationships will be presented in terms of the BED, calculated according to the following formula: BED = *D*(1 + *d*/(α/β)), where *D* is the total dose and *d* is the dose per fraction or alternatively, in terms of biologically equivalent dose in 2 Gy fractions (EQD2) calculated by using the formula: EQD2 = BED/(1 + 2/(α/β)).

Dose–response models according BED or EQD2 will be presented for normal tissue toxicity.

## Results

### Dose–Response Relationship for TCP

In Table 1, we list the papers (16–33) that focused on TCP modeling that were selected according to previously reported criteria. The chosen cell survival model and the type of curve used to fit/calculate the TCP is reported in the second and third columns, respectively. The means used in the paper to validate or test the model are shown in the last column, together with relevant additional information when needed [i.e., total number of patients either in internal cohorts (ICs) or in selected studies when published data (PD) were used].

**Table 1:** Tumor control probability (TCP) models derived for patients treated with SBRT.

Reference	Cellular model	TCP model	Validation	Patients
Chi et al. (16)	LQ	None	PD	1,224
Guckenberger et al. (17)	LQ, LQ-L	Logistic, Constant	IC	395
Guckenberger et al. (18)	LQ	Logistic	IC	796
Guerrero and Carlson (20)	LQ+Rep+Hyp	None	PD	0
Huang et al. (21)	LQ	Logistic, Gaussian	IS	
Klement et al. (22)	LQ	SVM	IC	399
Kong et al. (19)	LQ, Q	Logistic	PD	767
Lindblom et al. (23)	LQ, LQ-L+Hyp	Poisson, Logistic	IS	
Lindblom et al. (24)	LQ+Re+Rep+Hyp	Poisson, Logistic	IS	
Mehta et al. (25)	LQ, LQ-L	Logistic	PD	2,696
Ohri et al. (26)	LQ	Logistic	IC	482
Park et al. (27)	LQ-L	None	IV	
Ruggieri (28)	LQ+Rep+Hyp	Poisson	IS	
Ruggieri et al. (29)	LQ+Rep+Hyp	Poisson	IS	
Ruggieri et al. (30)	LQ+Rep+Hyp	Poisson	PD	246
Santiago et al. (31)	LQ, LQ-L	Logistic	PD	2,319
Strigari et al. (32)	LQ+Re+Rep+Hyp	Poisson	PD	1,095
	LQ-L+Re+Rep+Hyp			
Tai et al. (33)	LQ+Repopulation	Gaussian	PD	3,898

*LQ, linear quadratic; LQ-L, linear quadratic-linear; SVM, support vector machine; PD, published data; IC, internal cohort; IS, in silico; IV, in vitro; Re, repair; Rep, repopulation; Hyp, hypoxia*.

*The number in the last column refers to the patients used in the study*.

Most of published papers used the LQ model or the linear quadratic-linear (where the cell survival curve becomes linear above a threshold dose DT) model as tumor cell survival model with α/β ratio =10 Gy or higher.

Chi et al. (16) used the LQ model and reported that an α/β ratio >10 Gy may be more appropriate for dose–response prediction in SBRT of lung tumors.

Guckenberger et al. (17) compared the LQ and the LQ-L formalism in modeling local TCP in SBRT for stage I NSCLC. TCP showed a strong dose–response relationship, with only the exception of a sub-group of patients treated with single-fraction SBRT.

More recently, Guckenberger et al. (18) evaluated the variations in local TCP of SBRT treatments among lung metastases of different primary cancer sites and among primary NSCLC and secondary lung tumors. They observed a strong dose–response relationship in primary NSCLC and metastatic cohort but did not observe any statistically significant difference in the maximum planning target dose encompassing 90% of the TCP.

Kong et al. (19) found that smaller T stage (*p* < 0.001) and higher values of (total dose) × (dose per fraction) were associated with improved TCP when high-dose ablative radiotherapy was used for treating early-stage NSCLC.

Recently, Guerrero and Carlson (20) developed a radiobiological model that quantifies the reoxygenation effect for different fractionations and modeled the hypoxic fraction in tumors as a function of the number of radiation treatments in order to develop a simple analytical expression for a reoxygenation term in biological effect calculations.

Huang et al. (21) investigated the optimal fractionation schemes comparable to the most used dose schedule (4 × 12 Gy, empirically determined based on safety, efficacy, and minimal toxicity), using several TCP (the Martel model, Fenwick model, Webb–Nahum model, EUD-based model, and Nitin model) and NTCP [Lyman–Kutcher–Burman (LKB), Fenwick, and modified equivalent uniform dose (mEUD) model] taken from the literature.

Klement et al. (22) used a support vector machine (SVM) approach with a logistic model of TCP and found that BED at the isocenter (BEDISO) was a strong predictor and also the most frequently selected input feature for the SVM. The inclusion of FEV1 (i.e., the forced expiratory volume in 1 s) in a bivariate logistic model along with BEDISO, lead to a better description of the data but reduced significantly the area under curve.

Lindblom et al. (23) investigated potential effects of hypoxia and extreme hypofractionation on TCP in SBRT treatments. The authors found that in a schedule of three to five fractions, the doses required to achieve satisfying levels of TCP were considerably lower when local oxygenation variability was included in the model, as compared to the case of static oxygenation. Tumor repopulation has been also included in the above model in a subsequent paper by Lindblom et al. (24).

Mehta et al. (25) adopted the LQ and universal survival curve (USC) models to determine the parameters of a sigmoidal TCP as a function of BED. A TCP ≥90% was achieved with BED ≥159 and 124 Gy for the LQ and USC models, respectively. Dose-escalation beyond a BED of 159 Gy (equivalent to 53 Gy in 3 fractions at the isocenter) using the LQ model is not likely to yield any clinically significant gain in TCP but may result in severe toxicity.

Ohri et al. (26) proposed a TCP model for early-stage NSCLC where 2-year local control rate after hypofractionated SBRT is expressed as a function of BED and tumor diameter.

In Park (27), an alternative method for analyzing the effect of SBRT is investigated that introduces a USC leading a superior agreement with the experimentally measured survival curves in the ablative, high-dose range (>8–10 Gy) without losing the strengths of the LQ model around the shoulder. The USC provides an empirically and a clinically well-justified rationale for SBRT while preserving the strengths of the LQ model for conventional fractionated RT.

Ruggieri (28) studied the therapeutic ratio dependence on the number of fractions (*n*) for NSCLC radiotherapy using experimental data to model acute and chronic hypoxia. The optimum number of fractions depends on the rapidity of re-oxygenation of chronically hypoxic cells, almost independently of the size of both chronic and acute hypoxic areas. In a successive study, Ruggieri et al. (29) adopted their model to calculate the iso-TCP = 88% dose per fraction for *n* fractions (d88(*n*)). Calculated d88(*n*) decreased when the number of fractions increased and the product D88(*n*) = n*d88(*n*), exhibited a relative minimum around *n* = 8, suggesting the adoption of 6 ≤ *n* ≤ 10 instead of *n* = 3 in SBRT for small NSCLC tumors (30).

Interestingly, Santiago et al. (31) questioned whether, based on reviewed clinical NSCLC treatment outcome data, it would be possible to decide between LQ and LQ-L models, and came to the conclusion that both models could describe local tumor control after conventionally and hypofractionated irradiation and were robust methods for predicting clinical effects.

Strigari et al. (32) introduced a dependence on both total dose and dose per fraction into the re-oxygenation rate of hypoxic cells. The model has been fitted to the published clinical data on local control at 3 years to determine the functional form of such dependence. This enhanced model confirms a higher efficacy of SBRT treatments at intermediate doses per fraction as compared to extreme hypofractionation.

Tai et al. (33) reported that the regrowth model with an α/β around 16 Gy can be used to predict the dose–response of lung tumors treated with SBRT and that a BED of around 120 Gy saturates the TCP curve.

**Figure 2:**
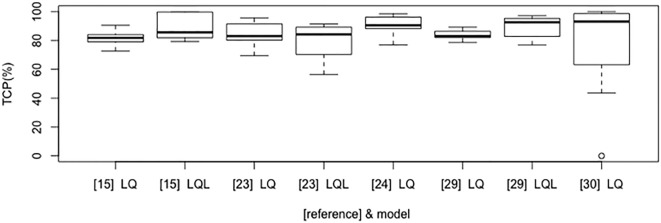
Boxplot that compares tumor control probability (TCP) results according to LQ/LQ-L-based selected models and published studies (in brackets).

Figure 2 shows the boxplot of TCP values for the most commonly investigated fractionation schedules (e.g., 25 Gy × 1 fraction; 12 Gy × 4 fractions; 15 Gy × 3 fractions; 7 Gy × 10 fractions; 6 Gy × 10 fractions; 30 Gy × 1 fraction; 12 Gy × 5 fractions; 34 Gy × 1 fraction; 18 Gy × 3 fractions; 20 Gy × 3 fractions) according to some of the selected models and their parameter sets. Of note, the investigated model and parameters are based on various endpoints (2 or 3 years TCP or crude TCP) that could affect the estimated outcome of the above schedules.

### Normal Tissue Complication Probability

Table 2 reports the papers (34–53) focusing on NTCP modeling selected according to the criteria specified in the Section “Materials and Methods.” The chosen cell survival model and the type of curve used to fit/calculate the NTCP for several toxicity endpoints is reported in the second and third columns, respectively. The means used in the paper to validate or test the model are shown in the last column, together with relevant additional information when needed (i.e., number of patients either in ICs or in selected studies when PD were used). In any case for normal tissues dose–effect relationships, most of the authors that reported dose–effect models used α/β = 3Gy with the exception of Wu et al. (43) who reports a wider range of α/β ratio.

**Table 2:** Normal tissue dose–toxicity models derived from patients undergoing SBRT.

Reference	Organs at risk	Toxicity	Cellular model	NTCP model	Validation	Patients
Avanzo et al. (34)	Lung	Severe acute radiological lung injury	LQ	Lyman EUD, logit EUD, relative seriality, population averaged critical volume model	IC	45
Guckenberger et al. (35)	Lung	Pneumonitis	LQ	Probit	IC	59
Grimm et al. (36)	Lung	≥G2 radiation pneumonitis (RP)	LQ	LBK	IC	18
Lee et al. (37)	Lung	Lung toxicity from 3 to 15 months post-SBRT	LQ	Lyman–Kutcher–Burman (LKB)	IC	21
Ricardi et al. (38)	Lung	≥G2 lung toxicity	LQ	logistic	IC	60
Wennberg et al. (39)	Lung	≥G2 RP	LQ, LQ-L	LKB	IC	57
Borst et al. (40)	Lung	≥ G2 RP	LQ	LKB	IC	128
Wang et al. (41)	Lung (mouse)	Death by Pneumonitis	LQ, LQ-L+Repair	None	PD	0
Nuyttens et al. (42)	Esophagus	G2 esophageal	LQ	LBK EUD	IC and PD	233
Wu et al. (43)	Esophagus	G2 acute esophageal	LQ	Logistic, Cox PH models	IC	125
Forquer et al. (44)	Brachial plexus	Brachial plexopathy	LQ-L	None	IC	253
Duijm et al. (45)	Bronchial structures	≥G1 (radiological)	LQ	Probit	IC	134
Karlsson et al. (46)	Bronchial structures	Atelectasia at 1,2,3 years	LQ–LQ-L	Lognormal accelerated failure time model,	IC	74
Xue et al. (47)	Major vessel^a^	G3-5 (aneurysm)	LQ	Logistic	IC and PD	625
Pettersson et al. (48)	Rib	Rib fracture	LQ	Logistic (with/without cut-off dose descriptor)	IC	68
Stam et al. (49)	Rib	Rib fracture	LQ	LKB EUD	IC	41
Stam et al. (50)	Rib	Rib fracture	LQ	LKB EUD	IC	494
Bongers et al. (51)	Chest wall	Chest wall pain	LQ	None	IC	500
Kimsey et al. (52)	Chest wall	≥G2 chest wall pain	LQ	Probit	IC	275
Woody et al. (53)	Chest wall	Chest wall pain	LQ	Logistic regression of mEUD and BMI	IC	102

*^a^Include any involved aorta, vena cava, pulmonary artery, or pulmonary vein*.

*LQ, linear quadratic; LQ-L, linear quadratic-linear; PD, published data; IC, Internal Cohort*.

*The number in the last column refers to the patients used in the study*.

### Normal Lung Toxicity

Avanzo et al. (34) investigated the early radiological radiation-induced lung toxicity comparing different NTCP models describing a SBRT cohort using the LQ model. They concluded that occurrence and severity depend on either dose or volume factor according to the chosen model.

Guckenberger et al. (35) used the LQ model with a probit curve to describe the incidence of pneumonitis as function of MLD.

Using the framework of the Lyman Model, Grimm et al. (36) estimated the risk of lung ≥G2 toxicity based on the analysis of clinical outcomes of SBRT treatments using a dose–response model against the total lung V20Gy and V5Gy for total, ipsilateral, and contralateral lung.

Lee at al (37) investigated the lung toxicity observed in six follow-up periods (from 3 to 15 months, at 3 monthly intervals) after SBRT and according tumor location. Interestingly, they reported the TD50, m and *n* parameters for six follow-up periods, dose calculation algorithm [analytical anisotropic algorithm (AAA) vs. Monte Carlo (MC) dose with convolution/superposition-based algorithms] and tumor location. The TD50 was significantly lower at 3 months after SBRT than at other time periods regardless of the dose calculation algorithm (i.e., AAA: 28 Gy; MC: 27 Gy). The threshold dose assessed at subsequent time points was not significant.

In the work of Ricardi et al. (38), the ≥G2 lung toxicity has been described using the LQ survival model and logistic NTCP approach against the MLD expressed as EQD2, obtaining values of TD50 and γ50 of 19.8 Gy and 2.2, respectively.

Wennberg et al. (39) investigated the ≥G2 radiation pneumonitisRP using both LQ and LQ-L model in the framework of LKB. The Fractional NTCP (NTCPfract) was obtained as [1-NTCP(Dx)/NTCP(Dx = 0)], where Dx is the dose lower than a cut-off level which is not taken into account in the calculation of NTCP. NTCPfract was calculated from the DVH of a representative patient with both USC or LQ corrected DVH data with NTCP parameters reported in Table 3.

**Table 3:** Model parameters for some normal lung tissue dose–toxicity models derived from patients undergoing SBRT, see Table 2 for additional information.

Reference	Model	TD50 (Gy)	*m*	*n*
Avanzo et al. (34)	LEUD	20.3	0.56	0.78
	LogEUD	18.3	3.91	0.84
	RS	21	0.84	0.42
Guckenberger et al. (35)	LQ	32.4	0.67	*
Lee et al. (37)	LQ COMSI > median	99.3	0.43	*
	LQ COMSI < median	89.3	0.33	*
Ricardi et al. (38)	LQ	24.5	0.18	0.87
Wennberg et al. (39)	USC	30	0.4	0.71
	LQ	30	0.4	0.87
Borst et al. (40)	LQ	19.6	0.43	1

*LQ, linear quadratic; Lyman-EUD (LEUD); Logit-EUD (LogEUD); relative seriality (RS); USC, universal survival curve; COMSI, the superior–inferior position of center-of-mass of planning target volume. TD50, tolerance dose at 50% probability of complication*.

*indicates that data are not reported.

Borst et al. (40) described a similar dose–response relationship for RP after hypofractionated SBRT and after the conversion of received dose to the equivalent EQD2 based on the LQ model with an α/β ratio of 3 Gy.

Based on published *in vitro* assays, alternative models such as the multi-target model (i.e., MTM) and the generalized linear quadratic model (gLQ) (41) have been tested against the standard LQ model. The gLQ equation was found superior to the LQ and MTM in predicting cell killing using SBRT.

**Figure 3:**
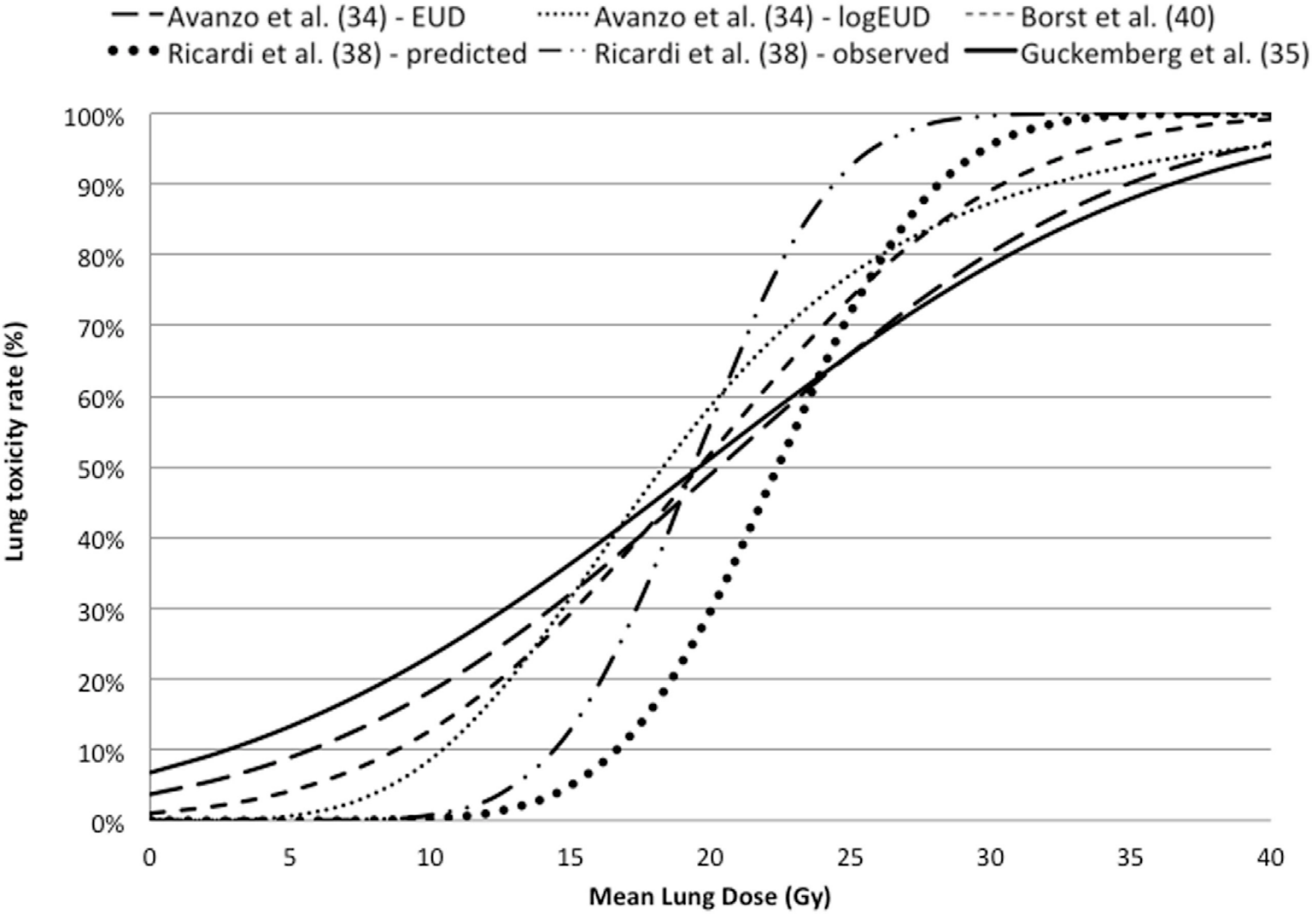
Dose–response curves for lung toxicity after SBRT against mean lung dose converted in equivalent dose in 2 Gy fractions using the linear quadratic model with an α/β ratio of 3 Gy.

The dose–response curves based on LQ model are reported against MLD in Figure 3, indicating that there is a good agreement in terms of TD50 and slope. More details of the estimated parameters can be found in Table 3.

### Esophageal Toxicity

Nuyttens et al. (42) described G2 esophageal toxicity using the LQ survival model and the LKB EUD model. Dose–response model for G2 esophagitis was reported for EUD, D10%, D5 cc, D1 cc, and Dmax in terms of 5-fraction equivalent dose using an α/β ratio of 3 Gy. The calculated TD50(V) (95% CI) and γ50 (95% CI) were 29.4 (26.9, 40.5) and 2.84 (1.11, 5.54) for EUD; 30.0 (26.7, 43.8) and 2.25 (0.97, 4.44) for D10%; 27.4 (23.0, 43.4) and 1.59 (0.81, 2.98) for D5cc; 32.9 (29.9,44.9) and 2.73 (1.10,5.75) for D1cc; and 43.4 (39.4,62.7) and 2.66 (1.02,5.40) for Dmax, respectively. Comparing data of two published dataset including the (42) for estimating a complication probability of 50% for grades 2 and 3 toxicity, the dose at 1cc (D1cc) resulted 32.9 and 50.7 Gy, respectively; while Dmax resulted 43.4 and 61.4 Gy, respectively.

**Figure 4:**
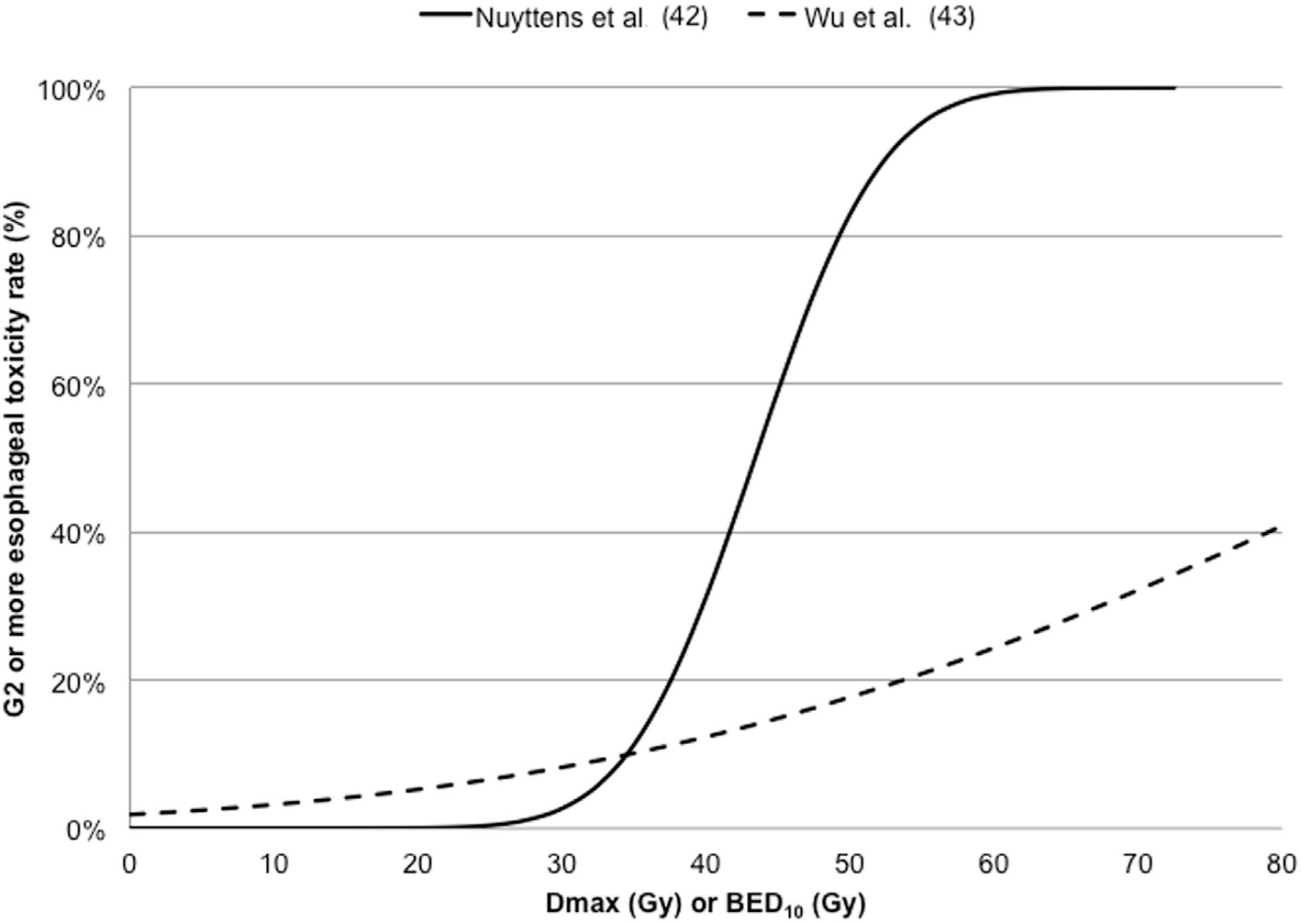
Dose–response curves for ≥ G2 esophageal toxicity against maximum dose (Dmax) converted in 5-fraction equivalent dose calculated using the linear quadratic (LQ) model with an α/β ratio of 3 Gy (42) or in BED_10_ calculated using the LQ model with an α/β ratio of 10 Gy (43).

Wu et al. (43) in a cohort of 125 patients investigated G2 acute esophageal using both Logistic and Cox PH models in the LQ survival framework with an α/β ratio of 10 Gy. The dose–response curves for G2 or more esophageal toxicity against Dmax are reported in Figure 4, indicating higher discrepancies at maximal doses higher than 40 Gy.

### Brachial Plexus

Forquer et al. (44) investigated the risk of brachial plexopathy using the LQ-L model in a large cohort of 253 patients. Although the same authors recommend to interpret carefully their results, as further late toxicity may develop after the extent of our current follow-up, they reported that LQ-L allows identifying a potentially more effective metric for doses per fraction over 6–7 Gy—typical of SBRT.

### Trachea and Bronchi

Duijm et al. (45) used the probit function and the LQ model to describe the ≥ G1 (radiological) toxicity of bronchial structures observed in 134 patients. The NTCP of the main/mid-/segmental bronchi according to Dmax, V65, V80, and V100 for the rate of adverse events (e.g., radiographically evident stenosis, occlusion, or atelectasis) has been reported. Authors reported for grade1 radiographically evident side effects, the 50% risk level for a 5-fraction schedule Dmax were 55 and 65 Gy for mid-bronchi and main stem bronchi, respectively. However, the same authors declared that their clinical toxicity could depend on many more factors, including patients’ fragility, than only radiation-induced side effects of the bronchi.

Karlsson et al. (46) compared the prediction of lognormal accelerated failure time model and the LQ or LQ-L survival model for dose correction in order to describe the bronchial structures atelectasia observed at 1, 2, and 3 years in a retrospective cohort of 74 patients with centrally located lung tumors treated with SBRT. A dose–response relationship between the incidence of atelectasis and the minimum dose to the high-dose volume of 0.1 cm^3^ of the bronchi was reported. Estimated incidence of radiation-induced atelectasis increased with dosage to 0.1 cm^3^ and time after treatment with an increased estimated incidence of atelectasis of up to 3% one-year post SBRT.

### Great/Major Vessels

Xue et al. (47) focused the study on major vessel toxicity in order to predict the radiation-induced aneurysm (G3-5) and used the logistic model in a large internal/external cohort within the LQ framework. Aorta dose–volume response model for V25 Gy, D4 cc, D1 cc, D0.5 cc, and Dmax have been calculated along with the DVH Risk Map, useful to compare literature constraints with predictions for an individual patient.

### Rib and Chest Wall

**Figure 5:**
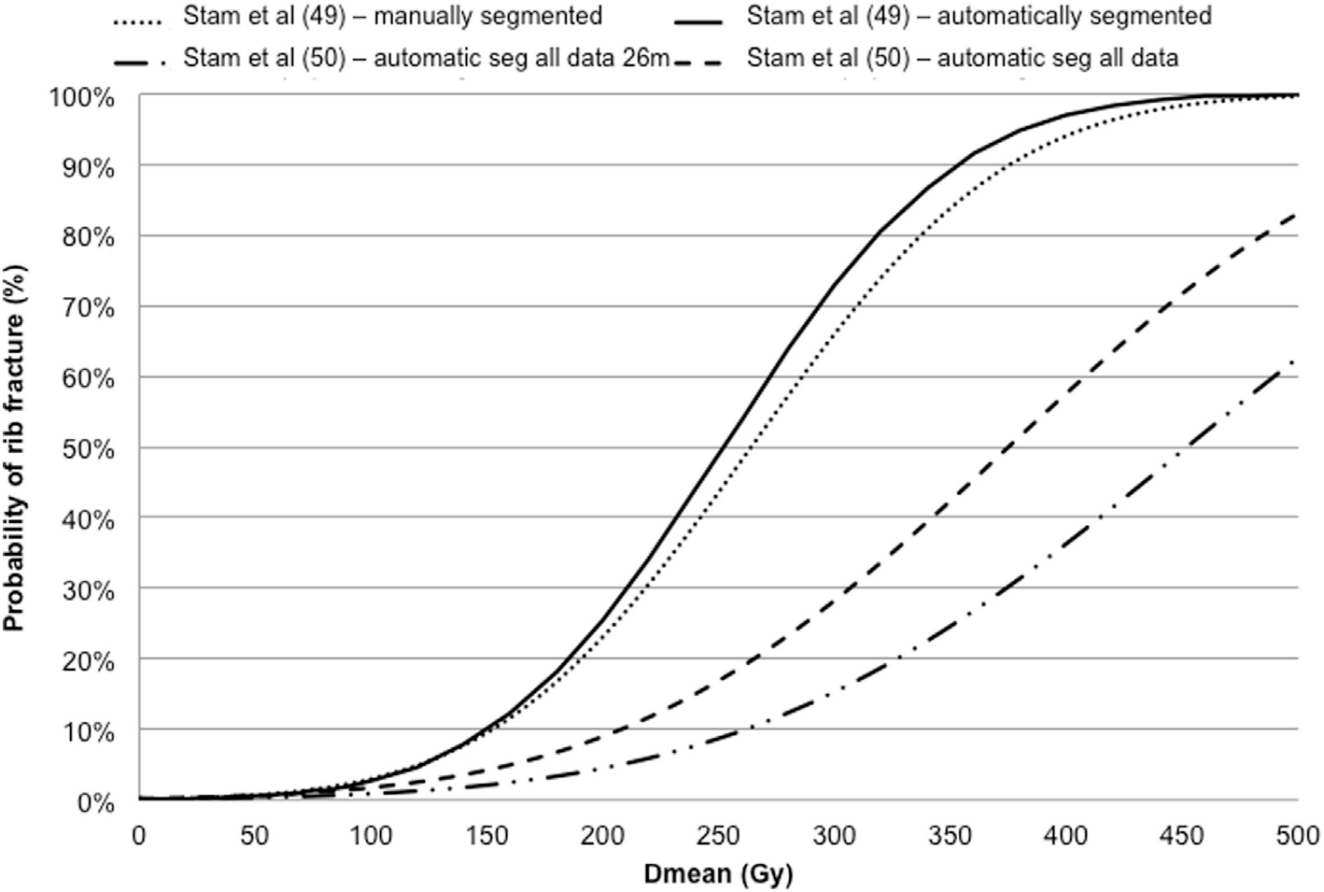
Dose–response models for rib fracture against the mean dose (Dmean) converted in biologically equivalent dose at 2 Gy fractions using the linear quadratic model with an α/β ratio of 3 Gy.

Pettersson et al. (48) described the observed risk of rib fracture using the LQ model with an α/β ratio of 3 Gy and the Logistic (with/without cut-off dose descriptor) function in a cohort of 68 patients. Rib fracture was investigated using the LQ survival model and LKB EUD NTCP model in Stam et al. (49, 50) investigating groups of 41 and 494 patients, respectively. The dose–response models for rib fracture for these two cohorts are reported against the mean dose converted to the biological equivalent dose at 2 Gy/fraction using the LQ model with an α/β ratio of 3 Gy (Figure 5). The optimal parameters for TD50 of Stam et al. (50) give values higher than previously reported (49) likely due to the fact that the first model does not take into account the time to toxicity. Moreover, whether all ribs, or only ribs that received high doses should be included in the NTCP model is a topic of debate as highlighted in the Supplementary Material of Ref. (50) and it could affect the dose constraints values to be adopted.

Regarding the chest wall pain, Bongers et al. (51) investigated the chest wall pain using the LQ on 500 patients; while Kimsey et al. (52) described the ≥G2 chest wall pain using the LQ model and probit functions on 275 SBRT patients. Finally, Woody et al. (53) described the chest wall pain observed in a cohort of 102 patients using the LQ model and the logistic regression approach including the mEUD as well as the patient body mass index.

## Discussion and Conclusion

With increasing application of SBRT in daily clinical practice, the identification of radiobiological models is necessary to prevent side-related complications caused by the generation of suboptimal treatment planning. The main issues are the robustness of used radiobiological models developed for commercial schedules when applied to non-standard fractionation.

### Robustness of Radiobiological Models

Due to the very limited experimental data, the validity of standard LQ model in hypofractionated radiotherapy is still debated (1, 54, 55). However, radiobiological models in the clinical setting are applied since they represent the unique strategy in the identification of clinical constraints of randomized SBRT trials in absence of further information specific for hypofractionated schedules. Indeed, most of RCTs report that constraints for a given treatment fractionation are based on known tolerance data, radiobiological conversion models, and the experience of several years of irradiation using these large fractions (RTOG 0236, RTOG 0618, RTOG 0915). However, in RTOG 0915, the capability of the LQ model to describe dose relation effects when the dose fraction is higher than 8 Gy has been questioned.

Our work highlights that the usefulness of radiobiological models and parameters obtained for SBRT treatments is increasing. Most of the identified models extract parameters based on institutional cohort without an external validation. Despite most models still do not have a validation on an independent cohort, the information extracted from SBRT series could represent a step forward to reinforce the appropriate application of a radiobiological model overcoming the limitation of using models derived for conventional fractionation series.

It should be stated that there are large uncertainties in the currently used biological models and associated parameters, so the application of results from conventional to SBRT approach (56) or from a dose algorithms to another (57) should be performed with caution.

Dose–volume constraints or biological model for SBRT optimization is especially hazardous when model parameters are derived for a conventional fractionation scheme. In absence of clinical data to provide guidance, the AAPM TG 166 (56) advises to adjust parameters (dose–volume or biological values) to steer critical organ doses into a dose–volume zone that is proven to be clinically safe.

### Investigated Endpoints of Radiobiological Models

Unfortunately, most of the models based on dose–toxicity relationships after SBRT are focused on different endpoints and are often based on a limited number of toxicity events with a potential frustration of modelers (58).

In addition, the adoption of appropriate dose-constraints and the introduction of modern image-based SBRT technology reduce the number of reported toxicity rate while demand further adjustments of models and related parameters or the development of new models describing novel induced side effects in different organs and tissues.

### Biologically Based Plan Optimization and Evaluation

It is important to emphasize that significant developments regarding optimization of treatment planning including physical and biological modeling started as soon as computers became available for treatment planning in the 1960s (59). Considering that the final dose distribution obtained using a physically- or biologically based optimization is in principle indistinguishable (59), the capability of biological-based optimization should be strongly considered as a potential advantage for planner or automatic systems when clinical plans are generated.

Even though dose–volume techniques are a mainstay of current clinical treatment planning optimization, biological optimization using complication probability models in intensity modulated [intensity-modulated radiotherapy (IMRT)] and volumetric arc [volumetric-modulated arc therapy (VMAT)] radiotherapy planning has shown potential for improved critical structure sparing also for SBRT treatment plan optimizations (6, 7, 60). Other authors noticed that there were minor or no dosimetric differences when gEUD objectives were used for fixed-beam IMRT and VMAT, likely due to small target volumes such as those encountered in SBRT for which fluence complexity is not as high as in large field intensity-modulated cases (61).

We would highlight that the awareness of increasing number of radiobiological modeling studies could help developing specific tool for the biologically based optimization of SBRT treatments. In this context, the objective function could incorporate dosimetric, biological, clinical, and technical considerations as well as uncertainties in measurements, calculations, and modeling to permit a true biological optimization and evaluation.

In particular, reliable indexes of radiobiological dose equivalency might facilitate the evaluation of dose–response relationships and plan comparison in multicenter trials or inter-institutional comparisons (62).

Concerning the use of radiobiological optimization in TPSs, this represents a useful option in various TPSs to be clinically used to plan patient treatments.

Although requiring further clinical validation, radiobiological modeling may prove to be a practical and convenient method for comparing different dose fractionation schemes.

### The Red Shell Concept

Given the differences in tumor size and location encountered in lung SBRT, some authors hypothesize that “one dose fractionation regimen does not fit all,” i.e., that there is a role for patient-specific dose prescription based on optimization of biological models in order to personalize the treatment planning (63). In this regard, Yang et al. (64) have introduced the LQ-based concept of “Red Shell.” This is a volume surrounding the clinical target volume where healthy tissues receive doses ranging from the prescription dose down to a threshold dose below which there is a low probability of undergoing late radiation damage. The extension of the Red Shell clearly depends on the α/β values of the involved (heterogeneous) normal tissues and on the chosen threshold probability/dose for the selected clinical endpoints. It is also suggested that the idea of defining a bounding surface inside which radiation damage is to be expected, need not to be limited to late effects only and that appropriate modeling can take into account acute effects as well.

### Isodose-Based Methodology

A method to generate isodose-based constraints and visually evaluate SBRT treatment plans, based on the published peer reviewed literature, has been reported in Ref. (65). In this work, the LQ model resulted to be valid up to a dose per fraction of 28 Gy and the α/β ratio was 2 for the spinal cord and brachial plexus, 4 for pneumonitis, 4 or 10 for acute skin reactions depending on treatment length, and 3 for late complications in other normal tissues.

### Ongoing Clinical Trials

Our understanding of the tumors and normal organs/tissues response to SBRT remains rather limited and should be improved based on the results of ongoing clinical trials. These results, obtained adopting radiobiologically based dose–volume constraints, allow radiobiological models to be stepwise refined and updated thus improving their validity, accuracy, and predictability (7, 66).

In addition, considering that SBRT is an extracranial departure from stereotactic radiosurgery with application in many different anatomic sites, there remain opportunities to advance each discipline through thoughtful attention to similarities and differences in their biologic impact on tumors and normal tissues. This is one of the aims of the ongoing AAPM Working Group on Stereotactic Body Radiation Therapy [AAPM Work Group SBRT (67)].

In addition, the adoption of validated and robust dose constraints may allow improving performance especially in Institutions with a limited experience in SBRT planning (68). Another potential issue of this study is related to the fact that the prescribed dose strongly varies from an Institute to another as has recently been described by Das et al. (69) reporting the State of Dose Prescription and Compliance to ICRU-83 in IMRT among Academic Institutions.

### Limitations and Challenges of Current Radiobiological Models

An important issue to be noted is that at doses per fraction higher than 10/16 Gy, it is commonly recognized that the success of traditional RT of bulky tumors is limited by several factors, such as poor blood flow, hypoxia, and toxicity, to the surrounding normal tissues. However, the radiobiology underlying SBRT modality may be distinct from that of conventional fractionation. In fact, recently published clinical results show that the LQ model actually underestimates tumor control by SBRT or SABR, likely because it does not take into account the significant contribution of indirect cell death and vascular damages. High-dose RT not only results in direct DNA damages, but it is also involved in non-targeted effects (70) including the out-of-field tumor response (i.e., abscopal and/or bystander effect), where the release of immune activating danger signals (apoptosis, necrosis, necroptosis) can induce anti-tumor immunity (or immunogenic forms of tumor cell demise). Indeed, the abscopal, bystander or non-target effect, can be regarded as irradiation-induced systemic anti-tumorigenic effects distant from the irradiated site in SBRT patients (71) and animal models (72). Moreover, several laboratory studies suggest that high dose/fractions (>8–10 Gy) may trigger additional biological effects (70–73) or processes, contributing to anti-tumor response and/or direct tumor cell killing. Further studies are mandatory to better clarify these aspects.

### Conclusion

Dose–volume constraints or biological model for SBRT optimization is especially hazardous when models and parameters are derived for a conventional fractionation schemes or different dose algorithm. Meanwhile, the high dose gradient of SBRT reduces the maximum dose per fraction on critical organs, thus allowing the application of radiobiological models with a reasonable reliability.

Robust models can be integrated in biological-based optimization tool, with a potential advantage for planner or automatic systems thanks to the control of a wide portion of dose–volume histogram although the superiority of biological-based optimization is still under debate. In addition, the final dose distributions obtained using a physical or biological-based optimization are in principle indistinguishable.

At the state of the art, it is still not possible to translate our ever-increasing knowledge of the underlying biological mechanisms into a prescription of the optimal radiation treatment plan without the application of radiobiological models which is currently adopted in RCTs or under validation in large prospective cohorts.

## Author Contributions

Conception or design of the work: LS. Acquisition, analysis, or interpretation of data for the work: LS, MD, SU, SS, AC, VB, and MB. Drafting the work or revising it critically for important intellectual content; LS, MD, SU, SS, AC, VB, and RM. Final approval of the version to be published: LS, MD, SU, SS, AC, VB, MB, and RM. Approval of final version: LS, MD, UU, SS, AC, VB, MB, and RM.

## Conflict of Interest Statement

The authors declare that the research was conducted in the absence of any commercial or financial relationships that could be construed as a potential conflict of interest.

## Abbreviations

BEDISO, BED at the isocenter; BED, biologically effective doses; EQD2, biologically equivalent dose in 2 Gy fractions; CTV, clinical target volume; CT, computed tomography; gLQ, generalized linear quadratic model; INDAR, individualized hyper-fractionated accelerated radiotherapy; IMRT, intensity-modulated radiotherapy; LQ, linear quadratic; LQ-L, linear-quadratic-linear; LKB, Lyman–Kutcher–Burman; MLD, mean lung dose; MDE, microscopic disease extension; mEUD, modified equivalent uniform dose; NSCLC, non-small cell lung cancer; NTCP, normal tissue complication probability; OAR, organ at risk; PTV, planning target volume; PET, positron emission tomography; PFTs, pulmonary function tests; RP, radiation pneumonitis; RPS, radiation pneumonitis requiring steroids; RCTs, randomized clinical trials; SABR, stereotactic ablative radiotherapy; SBRT, stereotactic body radiotherapy; SVM, support vector machine; CTVpath, total tumor-bearing region at pathologic examination; TCP, tumor control probability; USC, universal survival curve; VMAT, volumetric modulated arc therapy.
