# Supplementary material for: Radiobiological Optimization in Lung Stereotactic Body Radiation Therapy: Are We Ready to Apply Radiobiological Models?
Source: Front Oncol. 2018 Jan 8;7:321. doi: 10.3389/fonc.2017.00321 (PMC5766682; doi:10.3389/fonc.2017.00321)
Supplement: Supplementary file 1 [file Data_Sheet_1.PDF]

Supplementary material

We performed a literature search on PubMed using the following approach: (NSCLC) AND ((((((((NTCP NSCLC AND ("2000/01/01"[PDat] : "3000/12/31"[PDat]) AND Humans[Mesh])) OR ((((("tumor volume" or "local control") AND radiotherapy AND nsclc AND (modelling OR mathematical OR prediction)))) AND ("2000/01/01"[PDat] : "3000/12/31"[PDat]) AND Humans[Mesh])) OR (hypoxia TCP NSCLC AND ("2000/01/01"[PDat] : "3000/12/31"[PDat]) AND Humans[Mesh])) OR (microscopic extention NSCLC AND ("2000/01/01"[PDat] : "3000/12/31"[PDat]) AND Humans[Mesh])) OR (microscopic extention NSCLC AND ("2000/01/01"[PDat] : "3000/12/31"[PDat]) AND Humans[Mesh])) OR (microscopic TCP NSCLC AND ("2000/01/01"[PDat] : "3000/12/31"[PDat]) AND Humans[Mesh])) OR ((((("Carcinoma, Non-Small-Cell Lung"[Mesh] AND ("ratio" OR "Treatment Outcome"[Mesh] OR “models, statistical” [Mesh]) AND ("Radiation dosage" OR "Dose Fractionation"[Mesh]))) AND (model OR ratio))) AND ("2000/01/01"[PDat] : "3000/12/31"[PDat]) AND Humans[Mesh])) AND ("2000/01/01"[PDat] : "3000/12/31"[PDat]) AND Humans[Mesh]) Filters: From 2000/01/01, Humans
((NSCLC OR "Carcinoma, Non-Small-Cell Lung"[Mesh]) AND (SBRT OR "Radiosurgery"[Mesh] OR “stereotactic hypofractionated regimes”) AND((NTCP OR TCP OR “normal tissue complication” OR "tumor control" OR "Treatment Outcome"[Mesh] OR (TCP AND (hypoxic OR hypoxia)) OR (microscopic AND TCP)) AND (“probability” OR “rate” OR “models, statistical” [Mesh] OR radiobiology)), Filters: From 2000/01/01, Humans. The date of the last search was 1 October 2017.
